# Real-world prevalence of homologous recombination repair mutations in advanced prostate cancer: an analysis of two clinico-genomic databases

**DOI:** 10.1038/s41391-023-00764-1

**Published:** 2023-12-06

**Authors:** Irene M. Shui, Mehmet Burcu, Changxia Shao, Cai Chen, Chi-Yin Liao, Shan Jiang, Razvan Cristescu, Ravi B. Parikh

**Affiliations:** 1 Merck & Co., Inc., Rahway, NJ USA; 2 University of Wisconsin-Madison, Health Services Research in Pharmacy, Madison, WI USA; 3 Abramson Cancer Center, University of Pennsylvania, Philadelphia, PA USA; 4 Perelman School of Medicine, University of Pennsylvania, Philadelphia, PA USA

**Keywords:** Cancer epidemiology, Diagnostic markers, Cancer epidemiology, Prostate cancer

## Abstract

**Background:**

Homologous recombination repair mutation (HRRm) status may guide risk-stratification and treatment decisions, including polyadenosine diphosphate–ribose polymerase inhibitor use, in advanced prostate cancer. Although HRRm prevalence has been reported in single-institution studies or clinical trials, real-world HRRm prevalence in diverse populations is unknown. We describe HRRm in the clinical setting using two real-world clinicogenomic databases: the Flatiron Health and Foundation Medicine, Inc. Clinico-Genomic Database (CGDB), a national electronic health record-derived database, and the American Association for Cancer Research Project Genomics Evidence Neoplasia Information Exchange (GENIE).

**Methods:**

This cross-sectional analysis included 3757 individuals diagnosed with prostate cancer who had next generation sequencing (NGS) as standard of care. The CGDB included men with advanced/metastatic prostate cancer and genetic data included both germline and somatic pathogenic mutations. The GENIE analysis included men with prostate cancer whose received NGS as standard of care, but the data were filtered to include somatic mutations only. Due to key differences among databases, direct comparisons were not possible. Overall prevalence of HRRm was calculated and stratified by demographic and clinical characteristics.

**Results:**

HRRm prevalence (combined germline and somatic) in CGDB (*n* = 487) was 24.6% (95% CI 20.9–28.7%), with no major differences across demographic and disease characteristic subgroups. HRRm prevalence (somatic) in GENIE (*n* = 3270) was 11.0% (95% CI 10.0–12.1%), which varied between 9.5% and 18.4% across treatment centers.

**Conclusions:**

Approximately one-quarter of patients with advanced/metastatic prostate cancer in the CGDB had germline and/or somatic HRRm, which is consistent with clinical trials such as the PROfound study that used a similar NGS platform and algorithm to define HRRm. In the GENIE database, HRRm prevalence varied by treatment center or NGS platform. More research is needed to understand real-world HRRm prevalence variations.

## Introduction

Homologous recombination repair mutations (HRRms) in metastatic castration-resistant prostate cancer (mCRPC) are associated with aggressive disease and can indicate potential tumor susceptibility to polyadenosine diphosphate–ribose polymerase (PARP) inhibition [1, 2]. Recently, PARP inhibitors including olaparib and rucaparib have been approved to treat mCRPC [3–9]. Based on results of the phase III PROfound trial, olaparib was approved by the United States Food and Drug Administration (FDA) in 2020 as a treatment for patients with HRR-mutated mCRPC who progressed following prior treatment with enzalutamide or abiraterone [10, 11]. In the US prescribing information for olaparib, HRRm is defined as a pathogenic mutation in any of the following 14 genes: *BRCA1, BRCA2, ATM, BRIP1, BARD1,CDK12, CHEK1, CHEK2, FANCL, PALB2, RAD51B, RAD51C, RAD51D*, and *RAD54L* [11], which can be detected by the FDA-approved FoundationOne CDx [12]. Other testing by the FoundationOne Liquid CDx [13] and the BRACAnalysis CDx [14] were approved for the detection of *BRCA1/2* and *ATM*, and germline *BRCA1/2* mutations, respectively. In 2020, the US FDA also approved rucaparib as a treatment for patients with mCRPC associated with a deleterious germline and/or somatic *BRCA* mutation who were previously treated with androgen receptor–directed therapy and taxane-based chemotherapy [9] based on the results from the phase II TRITON2 trial [15]. Additionally, the phase III TRITON3 trial further showed that in patients with mCRPC with a *BRCA* mutation, the median duration of imaging-based progression-free survival was significantly longer with rucaparib compared with a physician’s choice control of docetaxel or a second-generation androgen receptor pathway inhibitor (abiraterone acetate or enzalutamide) [16].

Clinical trial evidence suggests that nearly a quarter of patients with mCRPC have tumors with DNA repair pathway gene mutations or alterations [2] Genetic testing to identify patients with HRRm is an essential tool to guide treatment in mCRPC. Several next-generation sequencing (NGS) platforms are used in real-world practice to determine HRRm status in mCRPC, including the FDA-approved tests previously mentioned to determine patient eligibility for olaparib treatment, as well as existing platforms that are standard of care at various institutions. Of note, FoundationOne CDx is the only companion diagnostic approved for detecting somatic and germline mutations in all 14 HRR genes indicated in the US prescribing information for olaparib [12]. In PROfound, the prevalence of any pathogenic mutation among those 14 genes was 26.9% in men whose tumors were successfully sequenced using the FoundationOne CDx assay [10]. However, clinical trial populations may not be representative of real-world populations, and there are incomplete data in the real-world clinical setting on overall HRRm prevalence defined by these 14 genes.

Accurate estimation of HRRm prevalence is key to identifying patients who may benefit from PARP inhibition monotherapy. Published data on HRRm prevalence in advanced prostate cancer derive from heterogeneous defintions of HRRm. For example, studies may assess prevalence in tumor versus germline samples or use different genes to define HRRm, and few studies have analyzed differences by patient demographics (e.g., state of disease or race/ethnicity) or testing panel [17–19]. The objectives of this study were (1) to describe real-world HRRm prevalence as defined by the 14 genes in the olaparib US label in advanced prostate cancer using two clinicogenomics databases, the American Association for Cancer Research (AACR) Project Genomics Evidence Neoplasia Information Exchange (GENIE) [20], and the Flatiron Health (FH) and Foundation Medicine, Inc. (FMI) Clinico-Genomic Database (CGDB) [21]; and (2) to understand how HRRm prevalence may vary by patient demographics, clinical characteristics, and treatment center.

## Materials and methods

### Study design and data sources

The CGDB [21, 22] is a de-identified longitudinal database originating from approximately 280 US cancer clinics (~800 sites of care). Retrospective longitudinal clinical data were derived from electronic health record data comprising patient-level structured and unstructured data, curated via technology-enabled abstraction, and were linked to genomic data derived from FMI comprehensive genomic profiling tests in the CGDB by de-identified, deterministic matching. The data used for this study were updated through December 31, 2020. GENIE [20] is an international cancer registry that provides clinical-grade, de-identified, next-generation cancer genomic sequencing data collected during routine medical practice. The data used in this analysis were from GENIE public version 10.1, which was updated through June 30, 2020.

### Patients

The CGDB patient cohort included men aged ≥18 years diagnosed with metastatic or advanced prostate cancer between January 1, 2018, and December 31, 2019, as assayed by FoundationOne CDx. Patients must have had a loss of heterozygosity score availability; at least two documented clinical visits at a site in the FH research network on or after January 1, 2011; and demographic information at FH and FMI that was uniquely and deterministically matched by a third party–linking vendor. Patients with histology not otherwise specified (e.g., not adenocarcinoma) were excluded [21, 22]. For the CGDB database, the data included age at sequencing, time between specimen collection and sequencing, race (White, Black, Asian, other, unknown or not collected), Gleason score and stage at initial diagnosis, Eastern Cooperative Oncology Group (ECOG) performance status, prostate-specific antigen (PSA) level, castration resistance or hormone sensitivity status, metastatic status at specimen collection date and at sequencing, practice type (community based, academic), and sample type (primary tumor, metastatic site). Ethnicity data were not available in the CGDB database. The GENIE cohort included men aged ≥18 years with prostate cancer who had NGS as standard of care between January 2011 and June 30, 2020. Patients with sample type not specified (e.g., primary vs. metastatic) were excluded. For the GENIE database, the data included age at sequencing, race (White, Black, Asian, other, unknown or not collected), ethnicity (non-Hispanic, Hispanic, unknown or not collected), sample type (primary tumor, metastatic site), and sequencing platform/treatment center (Dana-Farber Cancer institute [DFCI]; Duke Cancer Institute [Duke]; Memorial Sloan Kettering Cancer Center [MSK]; or other). Each of the three main centers used a different sequencing platform. DFCI and MSK each used a custom institutional panel, and Duke used a FMI panel; other sites used a mix of panels (Table S1). In addition to differences in the demographic and clinical variables available, a key difference between the datasets is in the type of mutations reported. The CGDB reports pathogenic mutations, regardless of somatic and germline origin, while filtering out most common benign germline mutations. Although the NGS performed on GENIE patient tumor tissue captured both germline and somatic mutations for their clinical care, ultimately germline mutation data were filtered out in the GENIE database for patient privacy. This filtering process has been previously described [23]. Although the filtering may not have removed all germline mutations (those with <0.0005% population frequency may remain), the HRRm mutations available for analysis in the GENIE dataset can be considered to be primarily somatic. Key differences between PROfound and the databases are listed in Table S2.

### Objectives and analyses

The primary objective was to determine the prevalence of HRRm based on the 14 genes indicated in the olaparib US prescribing information. The algorithm to determine HRRm from tissue samples was similar to that used in the PROfound trial [10, 24]. Only pathogenic or likely pathogenic gene alterations were included. DNA alterations were identified that result in truncation of the protein (nonsense mutations, frameshift mutations, and splice site mutations), large-scale (i.e., affecting at least a whole exon) genomic deletions/insertions/rearrangements, homozygous deletions, and other mutation types identified and reported as deleterious variants in the Breast Cancer Information Core database [25] or ClinVar [26]. Copy number alterations (e.g., genomic deletions/insertions and rearrangements, and homozygous deletions) were assessed for FoundationOne® CDx only due to difficulties in harmonizing copy number alteration calling across different NGS panels between AACR GENIE centers. Exploratory objectives were to describe the prevalence of mutations in *BRCA1* and *BRCA2* (jointly as *BRCA*m), *ATM*, and *CDK12*, and to understand how HRRm prevalence may vary by patient demographics, clinical characteristics, and treatment center. The analysis for this retrospective study was completed in April 2022. Due to key differences between databases, separate analyses were conducted for CGDB and GENIE.

### Statistical methods

Mean/standard deviation and median/interquartile range were calculated for continuous and count variables. Frequency and percentage were reported for categorical variables. Missing data were reported, and categories with low frequency or with small proportion were grouped together with other categories.

The overall prevalence (with 95% CIs) of HRRm was calculated as the number of patients with HRRm divided by the total number of study patients and was stratified by demographics, clinical characteristics, and NGS testing panels. In both databases, only one sample was analyzed from each patient. For patients with multiple samples, the most recent sample was selected if the results for each sample were concordant for HRRm, whereas the most recent positive sample was chosen for analysis of patients with discordant results. All analyses were descriptive, and no statistical comparisons were made. Due to the overall heterogeneity of available covariates in the two different databases and the lack of germline data for GENIE, no direct comparisons were performed between the cohorts.

### Study ethics

Institutional review board approval was not required for the CGDB or the GENIE databases because all personally identifiable characteristics had been intentionally omitted. Both FH-FMI and GENIE data were stored on a secure server owned by the study sponsor.

## Results

In the analysis, a total of 487 patients were included from CGDB and 3270 patients from GENIE. In CGDB, mean age at sequencing was 69.2 years (Table 1). Of the patients with data on race (*n* = 452), 70.8% were White, 10.2% were Black, 1.5% were Asian, and 17.5% did not fit into any of the above categories (other). Most patients (78.2%) in the CGDB database received primary oncology care at community-based practices. Most patients had high-risk disease (80.8% had a Gleason score of 8–10) and/or advanced disease (84.8% had stage IV disease) at diagnosis. Further, 77.4% of patients had metastatic disease and 17.2% had castration-resistant disease at the time of specimen collection. By the time sequencing occurred, these numbers increased to 97.9% and 51.1%, respectively. Of the 480 patients with data on sample type, 60.8% had a sample from the primary tumor and 39.2% from metastatic tissue (Table 1).

**Table 1:** CGDB patient characteristics.

	*N* = 487
Age at sequencing, mean (SD), years	69.2 (9.1)
Age at sequencing, *n* (%)	
<65 years	158 (32.4)
≥65 years	329 (67.6)
Time between specimen collection and sequencing, median (IQR), months	6.4 (1.4, 19.1)
Race^a^, *n* (%)	*n* = 452
White	320 (70.8)
Black	46 (10.2)
Asian	7 (1.5)
Other	79 (17.5)
Gleason score at initial diagnosis^b^, *n* (%)	*n* = 401
2 to 6	13 (3.2)
7	64 (16.0)
8 to 10	324 (80.8)
Stage at initial diagnosis^c^, *n* (%)	*n* = 363
I–II	32 (8.8)
III	23 (6.3)
IV	308 (84.8)
ECOG performance status at metastatic diagnosis^d^, *n* (%)	*n* = 357
0	181 (50.7)
1	142 (39.8)
2 or higher	34 (9.5)
PSA level at metastatic diagnosis^e^, median (IQR), ng/mL	41.7 (14.0, 216.0)
Castration-resistant at specimen collection, *n* (%)	
Yes	84 (17.2)
No	403 (82.8)
Metastatic disease at time of specimen collection, *n* (%)	
Yes	377 (77.4)
No	110 (22.6)
Castration-resistant at sequencing, *n* (%)	
Yes	249 (51.1)
No	238 (48.9)
Metastatic disease at sequencing, *n* (%)	
Yes	477 (98.0)
No	10 (2.1)
Practice type, *n* (%)	
Community	381 (78.2)
Academic	106 (21.8)
Sample type^f^, *n* (%)	*n* = 480
Metastasis	188 (39.2)
Primary (e.g., prostate)	292 (60.8)

*CGDB* Clinico-Genomic Database, *ECOG* Eastern Cooperative Oncology Group, *IQR* interquartile range, *PSA* prostate-specific antigen, *SD* standard deviation.

^a^Missing race information for 35 patients.

^b^Missing data on Gleason score at initial diagnosis for 86 patients.

^c^Missing data on stage at initial diagnosis for 124 patients.

^d^Missing data on ECOG performance status at metastatic diagnosis for 130 patients.

^e^Missing data on PSA level at metastatic diagnosis for 43 patients.

^f^Missing data on sample type for seven patients.

In the GENIE database, the mean age at sequencing was 66.9 years (Table 2). Patient characteristics were relatively consistent across treatment centers, with the exception of race and ethnicity. Of the 3270 patients in this database, 85.6% were White, 9.0% were Black, 3.4% were Asian, and 2.0% did not fit into any of the above categories (other). Between treatment centers, the lowest percentage of Black patients was at DFCI (5.2%) and the highest was at Duke (18.4%). Among 2987 patients with data on ethnicity, 95.3% were non-Spanish/non-Hispanic. DFCI and Duke had very few patients with Spanish/Hispanic ethnicity (1.1% and 0%, respectively) compared with MSK and other centers (5.1% and 11.2%, respectively) (Table 2). Across all sites, 62.6% of samples were from primary tumors and 37.4% were from metastatic tumors. Most patients (78.0%) were treated at MSK. Treatment centers used different NGS platforms; however, all platforms included in the analysis had coverage of at least 10 of the 14 genes, and only rare genes (with expected prevalence of <1%: *BRIP1, PALB2, BARD1, RAD51B, RAD54L, RAD51D, CHEK1, FANCL, RAD51C4* [10, 27]) were allowed to be missing (Table S1).

**Table 2:** GENIE patient characteristics.

Center	DFCI *n* = 369	Duke *n* = 142	MSK *n* = 2549	Other^a^ *n* = 210	Total *N* = 3270
Age at sequencing, mean (SD), years	67.5 (8.1)	67.8 (8.1)	66.7 (8.9)	67.2 (9.4)	66.9 (8.8)
Age group at sequencing, *n* (%)					
<65 years	139 (37.7)	51 (35.9)	1054 (41.3)	79 (37.6)	1323 (40.5)
≥65 years	230 (62.3)	91 (64.1)	1495 (58.7)	131 (62.4)	1947 (59.5)
Race, *n* (%)	*n* = 363	*n* = 136	*n* = 2397	*n* = 188	*n* = 3084^b^
White	334 (92.0)	110 (80.9)	2039 (85.1)	158 (84.0)	2641 (85.6)
Black	19 (5.2)	25 (18.4)	216 (9.0)	17 (9.0)	277 (9.0)
Asian^c^	5 (1.4)	1 (0.7)	93 (3.9)	6 (3.2)	105 (3.4)
Other	5 (1.4)	0 (0.0)	49 (2.0)	7 (3.7)	61 (2.0)
Ethnicity, *n* (%)	*n* = 369	*n* = 135	*n* = 2340	*n* = 143	*n* = 2987^d^
Non-Spanish/Non-Hispanic	365 (98.9)	135 (100)	2221 (94.9)	127 (88.8)	2848 (95.3)
Spanish/Hispanic	4 (1.1)	0 (0.0)	119 (5.1)	16 (11.2)	139 (4.7)
Sample type, *n* (%)					
Metastasis	191 (51.8)	70 (49.3)	891 (35.0)	72 (34.3)	1224 (37.4)

*COLU* Herbert Irving Comprehensive Cancer Center, Columbia University (New York, NY), *DFCI* Dana-Farber Cancer Institute (Boston, MA), *Duke* Duke Cancer Institute (Durham, NC), *GENIE* Genomics Evidence Neoplasia Information Exchange, *MSK* Memorial Sloan Kettering Cancer Center (New York, NY), *PHS* Providence Health & Services Cancer Institute (Portland OR), *UHN* Princess Margaret Cancer Center, University Health Network (Toronto, ON, Canada), *VICC* Vanderbilt-Ingram Cancer Center (Nashville, TN), *WAKE* Wake Forest Baptist Medical Center, Wake Forest University Health Sciences (Winston-Salem, NC), *YALE* Yale Cancer Center, Yale University (New Haven, CT).

^a^Other centers include COLU (*n* = 8), PHS (*n* = 20), UHN (*n* = 59), VICC (*n* = 43), WAKE (*n* = 12), and YALE (*n* = 68).

^b^Race of unknown/not collected (*n* = 186).

^c^Asian and non-Hispanic Asian included one patient that was originally recorded as Pacific Islander.

^d^Ethnicity of unknown/not collected (*n* = 283).

**Fig. 1:**
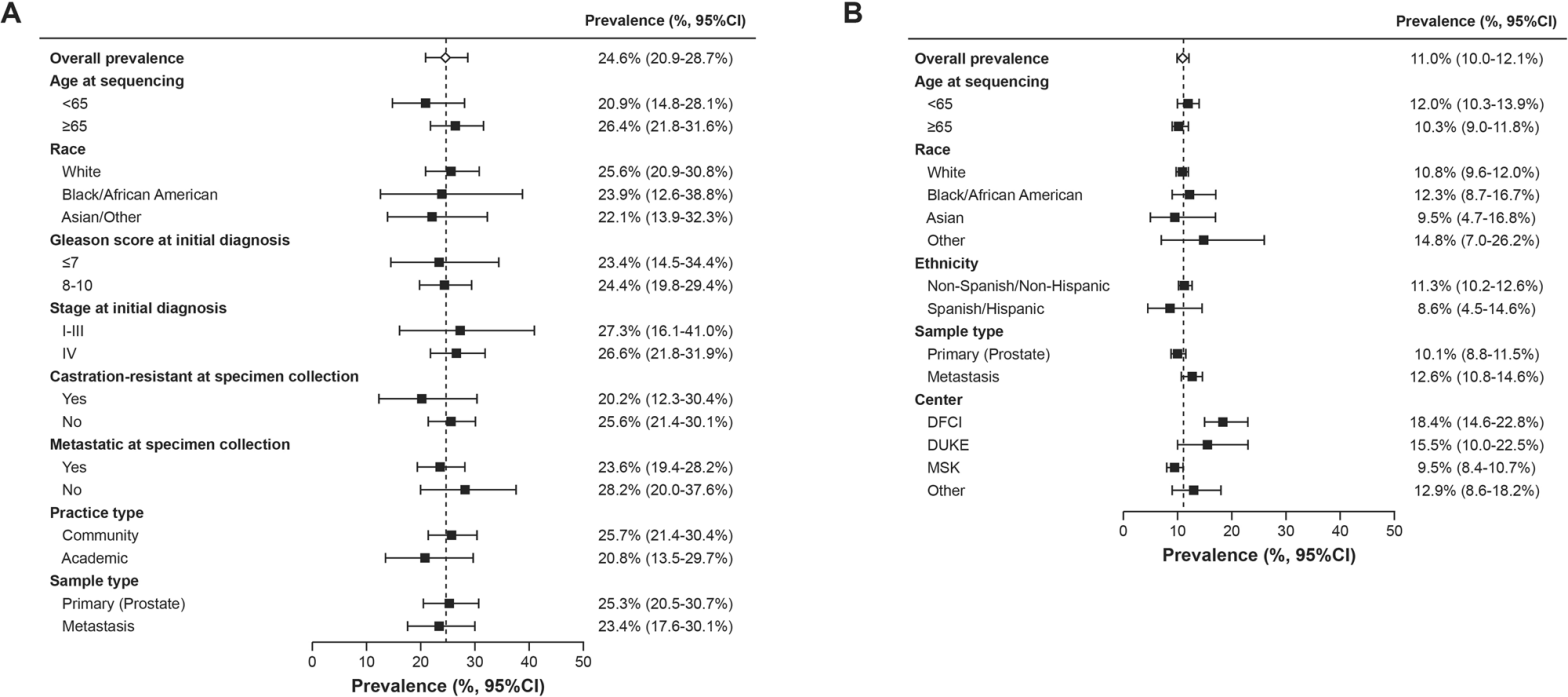
HRRm prevalence by selected characteristics. Estimates are listed with 95% confidence intervals. **A** CGDB. **B** GENIE. CGDB clinico-genomic database, GENIE Genomics Evidence Neoplasia Information Exchange.

The overall prevalence of HRRm was 24.6% in CGDB (somatic and germline) and 11.0% in GENIE (somatic only) (Table 3). The percent contributions for the individual gene components were relatively consistent for patients in PROfound [27] and FH-FMI-CGDB, with the most common HRRm component genes (*ATM*m*, BRCA*m, and *CDK12*m*)* contributing ~90% of the mutations (Table 3). No major differences were found in the overall HRRm prevalence by race in the CGDB data (Fig. 1A). In the GENIE database, HRRm prevalence varied by academic center (Fig. 1B). Patients treated at DFCI and Duke had higher HRRm prevalence (18.4% and 15.5%, respectively) compared with those treated at MSK (9.5%).

**Table 3:** HRRm prevalence and contribution by gene in PROfound, CGDB, and GENIE.

	PROfound [10, 27] (somatic and germline) *N* = 2792		CGDB (somatic and germline) *N* = 487		GENIE (somatic only)^a^ *N* = 3270	
	Prevalence, %	Contribution, %	Prevalence, % (95% CI)	Contribution, %	Prevalence, % (95% CI)	Contribution, %
*HRRm*^*b*^	26.9		24.6 (20.9–28.7)		11.0 (10.0–12.1)	
*ATM*	6.3	23.4	4.9 (3.2–7.2)	19.9	2.5 (2.0–3.1)	22.7
*BRCAm*	11.0	40.9	9.2 (6.8–12.2)	37.4	3.7 (3.1–4.4)	33.6
* BRCA1*	1.3	4.8	0.6 (0.1–1.8)	2.4	0.5 (3.0–8.3)	4.6
* BRCA2*	9.7	36.1	8.6 (6.3–11.5)	35.0	3.2 (2.6–3.8)	29.1
*CDK12*	7.1	26.4	7.4 (5.2–10.1)	30.1	4.3 (3.6–5.0)	39.1
*FANCL*	0.1	0.4	0.8 (0.2–2.1)	3.3	0 (---)	0
*BARD1*	0.4	1.5	0.6 (0.1–1.8)	2.4	0.1 (0–0.3)	0.9
*BRIP1*	0.5	1.9	0.6 (0.1–1.8)	2.4	0.2 (0.1–0.5)	1.8
*CHEK1*	0.1	0.4	0 (---)	0	0.1 (0–0.2)	0.9
*CHEK2*	1.6	6.0	1.9 (0.9–3.5)	7.7	0.3 (0.2–0.6)	2.7
*PALB2*	0.5	1.9	0.6 (0.1–1.8)	2.4	0.4 (0.2–0.7)	3.6
*RAD51B*	0.3	1.1	0.4 (0.1–1.5)	1.6	0.1 (0–0.3)	0.9
*RAD51C*	0.04	0.2	0 (---)	0	0 (---)	0
*RAD51D*	0.2	0.7	0 (---)	0	0 (---)	0
*RAD54L*	0.4	1.5	0.6 (0.1–1.8)	2.4	0.1 (0–0.3)	0.9

*CGDB* Clinico-Genomic Database, *GENIE* Genomics Evidence Neoplasia Information Exchange, *HRRm* homologous recombination repair mutation.

^a^GENIE filters out germline mutation for patient privacy reasons.

^b^Some patients had co-occurring mutations so the individual genes may not sum to the overall HRRm prevalence.

In the CGDB data, prevalence of mutations in the most common individual component genes (*BRCA*m, *ATM*m, and *CDK12*m) was also generally consistent across race- and ethnicity-based subgroups based on clinical characteristics (Table 4). However, in the GENIE database, there were suggestive differences in the prevalence of these genes between centers and race (Table 4). Specifically, the prevalence of *BRCA*m and *ATM*m was higher in patients treated at DFCI (6.8% and 6.5%, respectively) compared with patients treated at MSK (3.0% and 2.0%, respectively) and Duke (3.5% and 0.7%, respectively). The prevalence of *CDK12*m was also higher in patients treated at Duke (7.7%) compared with patients treated at DFCI (3.8%) and MSK (4.2%). White patients had higher prevalence of *BRCA*m (3.7%) and *ATM*m (2.7%) compared with Black patients (2.2% and 1.1%, respectively), whereas Black patients had higher prevalence of *CDK12m* (6.9%) compared with White patients (3.9%) (Table 4).

**Table 4:** CGDB and GENIE prevalence of *BRCA*m, *ATM*m, and *CDK12*m by selected characteristics.

CGDB				
	Sample size (*n*)	*BRCA*m % (95% CI)	*ATM*m % (95% CI)	*CDK12*m % (95% CI)
Overall prevalence	487	9.2 (6.8–12.2)	4.9 (3.2–7.2)	7.4 (5.2–10.1)
Age at sequencing				
<65 years	158	5.7 (2.6–10.5)	2.5 (0.7–6.4)	9.5 (5.4–15.2)
≥65 years	329	10.9 (7.8–14.8)	6.1 (3.8–9.2)	6.4 (4.0–9.6)
Race				
White	320	9.7 (6.7–13.5)	5.6 (3.4–8.7)	6.9 (4.4–10.2)
Black or African American	46	8.7 (2.4–20.8)	6.5 (1.4–17.9)	8.7 (2.4–20.8)
Asian and other race	86	9.3 (4.1–17.5)	2.3 (0.3–8.1)	7.0 (2.6–14.6)
Missing	35	5.7 (0.7–19.2)	2.9 (0.1–14.9)	11.4 (3.2–26.7)
Gleason score at initial diagnosis				
≤7	77	9.1 (3.7–17.8)	3.9 (0.8–11.0)	6.5 (2.1–14.5)
8–10	324	8.6 (5.8–12.2)	4.9 (2.8–7.9)	8.3 (5.6–11.9)
Unknown/not documented	86	11.6 (5.7–20.3)	5.8 (1.9–13.0)	4.7 (1.3–11.5)
Stage at initial diagnosis				
I-III	55	5.5 (1.1–15.1)	1.8 (0.0–9.7)	14.5 (6.5–26.7)
IV	308	9.1 (6.1–12.9)	5.8 (3.5–9.1)	8.4 (5.6–12.1)
Missing	124	11.3 (6.3–18.2)	4.0 (1.3–9.2)	1.6 (0.2–5.7)
Castration-resistant at specimen collection				
Yes	84	8.3 (3.4–16.4)	2.4 (0.3–8.3)	7.1 (2.7–14.9)
No	403	9.4 (6.8–12.7)	5.5 (3.5–8.1)	7.4 (5.1–10.5)
Metastatic at specimen collection time				
Yes	377	9.5 (6.8–13.0)	5.3 (3.3–8.1)	6.1 (3.9–9.0)
No	110	8.2 (3.8–15.0)	3.6 (1.0–9.0)	11.8 (6.4–19.4)
Practice type				
Community	381	10.8 (7.8–14.3)	4.7 (2.8–7.4)	7.9 (5.4–11.0)
Academic	106	3.8 (1.0–9.4)	5.7 (2.1–11.9)	5.7 (2.1–11.9)
Sample type				
Primary (prostate)	292	8.9 (5.9–12.8)	4.8 (2.6–7.9)	8.2 (5.3–12.0)
Metastasis	188	9.0 (5.4–14.1)	4.8 (2.2–8.9)	6.4 (3.3–10.9)
GENIE				
Overall prevalence	3270	3.7 (3.1–4.4)	2.5 (2.0–3.1)	4.3 (3.6–5.0)
Age group at sequencing				
<65	1323	4.2 (3.1–5.4)	2.6 (1.8–3.7)	4.9 (3.8–6.2)
≥65	1947	3.3 (2.6–4.2)	2.4 (1.7–3.1)	3.8 (3.0–4.7)
Race				
White	2641	3.7 (3.0–4.5)	2.7 (2.1–3.4)	3.9 (3.2–4.7)
Black	277	2.2 (0.8–4.7)	1.1 (0.2–3.1)	6.9 (4.2–10.5)
Asian^a^	105	2.9 (0.6–8.1)	0.0 (0.0–3.5)	6.7 (2.7–13.3)
Other	61	6.6 (1.8–15.9)	1.6 (0.0–8.8)	6.6 (1.8–15.9)
Unknown/not collected	186	4.8 (2.2–9.0)	2.7 (0.9–6.2)	3.2 (1.2–6.9)
Ethnicity				
Non-Spanish/non-Hispanic	2848	3.8 (3.1–4.6)	2.5 (2.0–3.1)	4.5 (3.8–5.3)
Spanish/Hispanic	139	3.6 (1.2–8.2)	1.4 (0.2–5.1)	3.6 (1.2–8.2)
Unknown/not collected	283	2.5 (1.4–5.0)	2.8 (1.2–5.5)	2.1 (0.8–4.6)
Sample type				
Primary	2046	3.6 (2.8–4.5)	2.3 (1.7–3.0)	3.7 (2.9–4.6)
Metastasis	1224	3.8 (2.8–5.1)	2.8 (1.9–3.9)	5.1 (4.0–6.5)
Center				
DFCI	369	6.8 (4.4–9.8)	6.5 (4.2–9.5)	3.8 (2.1–6.3)
Duke	142	3.5 (1.2–8.0)	0.7 (0.0–3.9)	7.7 (3.9–13.4)
MSK	2549	3.0 (2.4–3.8)	2.0 (1.5–2.6)	4.2 (3.4–5.0)
Other^b^	210	6.2 (3.3–10.4)	2.9 (1.1–6.1)	3.8 (1.7–7.4)

*CGDB* Genomic Database. COLU, Herbert Irving Comprehensive Cancer Center, Columbia University (New York, NY), *DFCI* Dana-Farber Cancer Institute (Boston, MA); Duke, Duke Cancer Institute (Durham, NC), *GENIE* Genomics Evidence Neoplasia Information Exchange, *MSK* Memorial Sloan Kettering Cancer Center (New York, NY), *PHS* Providence Health & Services Cancer Institute (Portland, OR), *UHN* Princess Margaret Cancer Center, University Health Network (Toronto, ON, Canada), *VICC* Vanderbilt-Ingram Cancer Center (Nashville, TN), *WAKE* Wake Forest Baptist Medical Center, Wake Forest University Health Sciences (Winston-Salem, NC), *YALE* Yale Cancer Center, Yale University (New Haven, CT).

^a^Includes one patient who was originally recorded as Pacific Islander.

^b^Other center includes COLU, PHS, UHN, VICC, WAKE, and YALE.

## Discussion

Approximately one-quarter of patients with advanced/metastatic prostate cancer in the CGDB database with relevant testing data available had tumors with HRRm. This prevalence in our real-world study was consistent with the findings from the PROfound trial, which used a similar NGS platform and algorithm to define HRRm [10, 27]. Further, the contribution of the 14 component genes was also relatively consistent between the CGDB data and PROfound, especially for the most common component genes (*BRCA*m*, ATM*m, and *CKD12*m). When the prevalence of HRRm was analyzed by various patient characteristics, no major differences were observed in the CGDB database; however, the sample sizes stratified by patient groups were small (46 African American and seven Asian patients), and no data on ethnicity were available.

Direct comparisons between GENIE and PROfound or FH-FMI-CGDB were not possible as the GENIE database filtered germline mutations, whereas pathogenic germline mutations were retained in FM-FMI-CGDB. The breakdown of somatic and germline prevalence for the PROfound or the FH-FMI-CGDB databases was not available. In addition, the contribution of germline and somatic mutations can vary for the different component HRRm genes. For example, *BRCA2* studies have shown the prevalence in mCRPC to range from 3.3% to 6.0% and 5.0% to 15.1% for germline and somatic mutations, respectively [28]. Estimates from studies have shown that approximately 36% to 52% of *BRCA2* mutations were predicted to be germline, whereas *CDK12* mutations are almost always somatic [29–31].

The large sample size in the GENIE database (*n* = 3270) and the diversity of patients and treatment centers allowed for the assessment of differences in somatic HRRm prevalence based on the treatment center and race. We observed differences in HRRm prevalence by treatment center in the GENIE database. Several possible factors may have contributed to the differences between centers. First, different NGS platforms were used to detect mutations (Table S1). The NGS platform used by MSK is a tumor normal-based platform that may filter out germline mutations more efficiently compared with the tumor-only platforms used at DFCI and Duke, which might contribute to the lower overall HRRm prevalence observed at MSK. None of the NGS panels for this study were missing more than 4 genes; given that the missing genes had <1% expected prevalence, this likely did not contribute significantly. For example, the lowest prevalence of HRRm was found in patients treated at MSK whose NGS platform was only missing *FANCL*, which had a prevalence of 0.1% in the PROfound trial [10, 27]. Different platforms may also have variable sensitivity to detect specific variants and/or variable postprocessing bioinformatics variant calling algorithms. For example, *CDK12m* is predominantly a somatic mutation that would not be affected by germline filtration [30], but we still observed differences in prevalence by treatment center/NGS platform. Another study by Armenia et al. [32] has presented data on *CDK12m* prevalence that varied by NGS platform (5% prevalence in MSK-Impact data vs 11% prevalence in FMI data).

Variations in the racial composition of patient groups at each site might also have contributed to the observed differences in HRRm by treatment center. The prevalence of *CKD12m* was higher in patients at Duke (7.7%) compared with patients from MSK (4.2%) and DFCI (3.8%). We observed that Black patients had higher prevalence of *CDK12m* compared with White patients (6.9% vs 3.9%) and that Duke had a higher proportion of Black patients (18.4%) compared with DFCI (5.2%) and MSK (9.0%).

Even though no differences were found in overall HRRm prevalence by race, differences in *BRCA*m*, ATM*m, and *CDK12*m by race were observed. There has been a lack of diversity in most advanced/metastatic prostate cancer cohorts and variable definitions of component genes in previously published literature. Two prior studies examined the overall genomic landscape among patients from GENIE, but this analysis was limited to MSK and DFCI data [33, 34]. In one study among patients with metastatic prostate cancer (*n* = 909), Black men were more likely than White men to have actionable mutations overall, specifically in the DNA repair pathway genes (as defined by *ERCC5, MRE11, TP53BP1, POLE, RAD21, MSH2, MSH6, BRCA1/2, ATR*, and *ATM*). The frequency of *CDK12*m was also higher in Black versus White men with metastatic prostate cancer. Differences in race were less pronounced in patients with primary tumor samples [33]. The other study used a similar cohort but removed 458 duplicate samples and did not find a statistically significant difference in DNA repair mutations between Black and White men [34]. Compared with these previous GENIE analyses, our study used updated data, included all contributing centers with relevant NGS platforms, and focused on the HRRm definition specific to olaparib, which resulted in a larger number of Black men included in the current study (*n* = 277 compared with *n* = 71 reported by Mahal et al. [33] and *n* = 77 reported by Schumacher et al. [34]). In a study of 2069 men, including 169 Black men, with prostate cancer, genomic differences by race were found using MSK-IMPACT data [29]. Tumors from Black men harbored fewer phosphatase and tensin homolog mutations and more androgen receptor alterations than tumors from White men, and tumors from Asian men had more forkhead box A1 mutations and more zinc finger homeobox 3 alterations than White men. in our study, no differences were observed in overall DNA repair alterations by race, but the definitions for HRRm between our study and the previous studies were inconsistent [29]. Another study compared the prevalence of pathogenic/likely pathogenic germline variants in Black and White men with metastatic prostate cancer and found that Black men were more likely to have a germline *BRCA1* mutation and were less likely to have a non-*BRCA* DNA repair germline variant (as defined by *MSH2, MSH6, PMS2, MLH1,ATM, RAD50, RAD51D, NBN, CHEK2, BRIP1, PALB2, RAD51C, ATM, BLM*, and *TP53*) [35]. Note that there were too few *BRCA1* mutations (0.5%) among somatic mutations in our study to assess differences by race. More research in diverse populations is needed to confirm whether these differences truly exist and are clinically significant.

### Study limitations

The GENIE database mostly represents major academic centers, whereas the CGDB database is mostly community based. Although complementary, these databases may not be generalizable to the overall real-world population of patients with advanced prostate cancer. For example, the CGDB patient cohort was predominantly stage IV at initial diagnosis, and at the time of sequencing, 98% had metastatic disease and 51.1% had castration-resistant disease. GENIE provided limited patient clinical data, and no information is available about the Gleason score and stage at diagnosis or whether patients had metastatic or castration-resistant disease at the time of sequencing [20]. Furthermore, databases were limited to patients receiving NGS testing as standard of care. Although the National Comprehensive Cancer Network guidelines for prostate cancer began recommending HRRm testing for patients with metastatic prostate cancer in 2019 [36], a survey of providers treating advanced prostate cancer found that in early 2020, only 38% of US patients with mCRPC were tested for HRRm [37]. In another study using data from 2014 to 2018 (prior to the approval of PARP inhibitor for prostate cancer), only 13% of patients with mCRPC were tested for HRRm [19]. In addition, because the GENIE database filters out most germline mutations, we were unable to determine the total prevalence of HRRm in this population and could not make direct comparisons with CGDB or PROfound. However, the large and diverse GENIE database provided the opportunity to assess differences by testing center and race. Finally, data represent prevalence among those who were tested in real-world practice that may represent potential selection bias.

## Conclusion

In summary, the CGDB data have shown that the prevalence of HRRm in real-world clinical data (24.6%) was consistent with the prevalence of HRRm in the PROfound trial (26.9%) when a similar NGS platform (FoundationOne CDx) and algorithm were used. When testing was performed using different NGS platforms (GENIE database), HRRm prevalence was variable across treatment centers. Suggestive racial differences were observed for the most common HRRm genes, but not in overall HRRm prevalence. Because Black men have the highest incidence of and mortality from prostate cancer, more studies with increased diversity in genomic testing cohorts are urgently needed. To our knowledge, this is the first and largest analysis to provide HRRm prevalence data defined by 14 different genes using a definition consistent with the olaparib indication and to assess differences by patient characteristics and treatment center/NGS platforms.

## Supplementary information

Supplemental Material

## Data Availability

The data that support the findings of this study originated by Flatiron Health, Inc. Requests for data sharing by license or by permission for the specific purpose of replicating results in this manuscript can be submitted to publicationdataaccess@flatiron.com. With agreement to terms of access, AACR Project GENIE Data is currently available via two mechanisms: 1. Synapse Platform(SageBionetworks): https://synapse.org/genie. 2. cBioPortal for Cancer Genomics (MSK): https://www.cbioportal.org/genie/.

## Code availability

Data analysis was conducted using SAS Studio 3.8 running 9.4 TS1 M6 and R statistical software. Contact the study team for access to the study code.
