# Supplementary material for: Real-world prevalence of homologous recombination repair mutations in advanced prostate cancer: an analysis of two clinico-genomic databases
Source: Prostate Cancer Prostatic Dis. 2023 Dec 6;27(4):728–35. doi: 10.1038/s41391-023-00764-1 (PMC11543596; doi:10.1038/s41391-023-00764-1)
Supplement: Supplementary file 1 — Supplemental Material [file 41391_2023_764_MOESM1_ESM.docx]

**Supplemental Material**

**Table S1.** NGS Panels Usable for Testing HRRm-Associated Genes

| **Treatment Center** | **Panels** | **Number of HRRm-associated genes covered** | **Gene(s) missing^b^** |
| --- | --- | --- | --- |
| COLU | COLU-CCCP-V1 | 13 | FANCL |
| DFCI | DFCI-ONCOPANEL-3 | 12 | RAD54L, RAD51B |
|  | DFCI-ONCOPANEL-3.1 | 12 | RAD54L, RAD51B |
| Duke | DUKE-F1-T7^a^ | 14 | none |
|  | DUKE-F1-DX1^a^ | 14 | none |
| MSK | MSK-IMPACT341  MSK-IMPACT410  MSK-IMPACT468 | 13  13  13 | FANCL  FANCL  FANCL |
| PHS | PHS-TRISEQ-V2 | 10 | BARD1, RAD51B, RAD51C, RAD51D |
|  | PHS-TST170-V1 | 14 | none |
| UHN | UHN-555-V1  UHN-555-PROSTATE-V1  UHN-OCA-V3 | 12  12  10 | RAD54L, RAD51B  RAD54L, RAD51B  BRIP1, BARD1, RAD54L, FANCL |
| VICC | VICC-01-T7^a^  VICC-01-T5A^a^  VICC-01-DX1^a^ | 14  14  14 | none  none  none |
| WAKE | WAKE-CLINICAL-T7 | 10 | RAD51B, RAD51C, RAD51D, RAD54L |
|  | WAKE-CLINICAL-DX1^a^ | 14 | none |
| YALE | YALE-OCP-V3 | 10 | BRIP1, BARD1, RAD54L, FANCL |

Abbreviations: COLU, Herbert Irving Comprehensive Cancer Center, Columbia University (New York, NY); DFCI, Dana-Farber Cancer Institute (Boston, MA); Duke, Duke Cancer Institute (Durham, NC); HRRm, homologous recombination repair mutation; MSK, Memorial Sloan Kettering Cancer Center (New York, NY); NGS, next-generation sequencing; PHS, Providence Health & Services Cancer Institute (Portland, OR); UHN, Princess Margaret Cancer Centre, University Health Network (Toronto, ON, Canada); VICC, Vanderbilt-Ingram Cancer Center (Nashville, TN); WAKE, Wake Forest Baptist Medical Center, Wake Forest University Health Sciences (Winston-Salem, NC); YALE, Yale Cancer Center, Yale University (New Haven, CT).

^a^These sequencing platforms are equivalent to the FoundationOne^®^CDx.

^b^From the PROfound trial prevalence of rare genes: *BRIP1* 0.5%, *PALB2* 0.5%, *BARD1* 0.4%, *RAD51B* 0.4%, *RAD54L* 0.4%, *RAD51D* 0.2%, *CHEK1* 0.1%, *FANCL* 0.1%, and *RAD51C* 0.04%.

**Table S2.** Key Comparisons Between the PROfound Trial and Study Databases

|  | **PROfound**^1,2^ | **CGDB** | **GENIE** |
| --- | --- | --- | --- |
| **Patients, n** | 2,792 | 487 | 3,720 |
| **Date of completion/ data update** | June 4, 2019 | December 31, 2020 | June 30, 2020 |
| **Assay** | FoundationOne^®^ CDx | FoundationOne^®^ CDx | NGS SOC^a^ |
| **Mutations captured** |  |  |  |
| Somatic | Yes | Yes | Yes |
| Germline | Yes | Yes | No |

CGDB, Clinico-Genomic Database; GENIE, Genomics Evidence Neoplasia Information Exchange; NGS, next-generation sequencing; SOC, standard of care.

^a^See table S1 for more information.

**References for Supplemental Material**

1. de Bono J, Mateo J, Fizazi K, et al: Olaparib for metastatic castration-resistant prostate cancer. N Engl J Med 382:2091-2102, 2020
2. de Bono JS, Fizazi K, Saad F, et al: Central, prospective detection of homologous recombination repair gene mutations (HRRm) in tumour tissue from >4000 men with metastatic castration-resistant prostate cancer (mCRPC) screened for the PROfound study. Ann Oncol 30:v328-v329, 2019. Abstract 5118
